# High-throughput neuro-imaging informatics

**DOI:** 10.3389/fninf.2013.00031

**Published:** 2013-12-17

**Authors:** Michael I. Miller, Andreia V. Faria, Kenichi Oishi, Susumu Mori

**Affiliations:** ^1^Center for Imaging Science, Johns Hopkins Whiting School of Engineering, The Johns Hopkins UniversityBaltimore, MD, USA; ^2^Institute for Computational Medicine, Johns Hopkins School of Medicine and Whiting School of Engineering, The Johns Hopkins UniversityBaltimore, MD, USA; ^3^Department of Biomedical Engineering, The Johns Hopkins UniversityBaltimore, MD, USA; ^4^The Russell H. Morgan Department of Radiology and Radiological Science, The Johns Hopkins University School of MedicineBaltimore, MD, USA

**Keywords:** neuro-imaging, neuroinformatics, computational anatomy, functional imaging

## Abstract

This paper describes neuroinformatics technologies at 1 mm anatomical scale based on high-throughput 3D functional and structural imaging technologies of the human brain. The core is an abstract pipeline for converting functional and structural imagery into their high-dimensional neuroinformatic representation index containing O(1000–10,000) discriminating dimensions. The pipeline is based on advanced image analysis coupled to digital knowledge representations in the form of dense atlases of the human brain at gross anatomical scale. We demonstrate the integration of these high-dimensional representations with machine learning methods, which have become the mainstay of other fields of science including genomics as well as social networks. Such high-throughput facilities have the potential to alter the way medical images are stored and utilized in radiological workflows. The neuroinformatics pipeline is used to examine cross-sectional and personalized analyses of neuropsychiatric illnesses in clinical applications as well as longitudinal studies. We demonstrate the use of high-throughput machine learning methods for supporting (i) cross-sectional image analysis to evaluate the health status of individual subjects with respect to the population data, (ii) integration of image and personal medical record non-image information for diagnosis and prognosis.

## Introduction

Imaging is one of the most powerful medical tools for monitoring human health. In the era of personalized medicine, periodic checkups via whole body imaging, combined with routine medical screening, genetic information, and comparison with population data is expected to be key information for monitoring health status, pathological condition, and therapeutic effect. High-throughput imaging technologies are becoming ubiquitous, driven by the deployment of whole body high resolution MR, CT, and PET imaging devices. While huge personal MR/CT based data records are routinely being collected for cross-sectional and longitudinal examination of the progression of diseases as manifest via tumor growth or atrophic neurodegeneration, currently while this information is stored in the medical PACS, usually only linguistic diagnostic encoding from the physician is stored in the searchable patient record. Such a lack of direct feature representation of the dense structural and functional phenotype precludes its use for systematic medical analysis such as population statistics or cross-modality correlation. Contrast this to what is emerging in high-throughput genomics.

There are several reasons. Clearly, utilizing the information from dense imagery from a longitudinal study, for example, presents daunting challenges. High-resolution whole body CT scans at 0.5 mm resolution for full body coverage would generate gigabytes of data. Visual inspection by a radiologist is overwhelming at the original resolution. Most often the images are down-sampled or low-resolution images are acquired to accommodate the storage and retrieval challenges. Constructing a parsimonious encoding of the discriminating information presents a fundamental challenge. In high-dimensional spaces such as that represented by the millions of measurements generated by 3D imagers, parsimonious representation of the measurable structural and functional phenotype is essential.

Exploiting the maximum potential of the imagers or the associated scans appears impractical without some form of encoding, or extreme data reduction. Reduction of high-dimensional imagery to symbolic knowledge representations encoded via the informative discriminating dimensions is one of the holy-grails of image analysis, a field which has advanced dramatically in the past several decades. From our own school of Grenander's metric pattern (Grenander, 1993) has emerged the field of computational anatomy (CA) for medical image analysis (Grenander and Miller, 1998, 2007; Toga and Thompson, 2001; Miller et al., 2002; Thompson and Toga, 2002; Ardekani et al., 2009; Ashburner, 2009; Pennec, 2009). The organizing principle in CA is that while there are variations in human structure and function, representation of the evolutionarily stable organization of processing in human beings are to a great extent organized around the structural manifestation of the genotype, throughout what we term the structural or anatomical phenotype. The evolutionary process has been masterful in its conservation of neural processing and its apparent organization around the macroscopic scales of human anatomy. We assume throughout that while functional layout is highly variable and ultimately associated with cellular architecture, it is manifest at the macroscopic scale of the topological organization of human anatomy and is preserved in large part cross-sectionally. Striking examples include the tonotopic organization of the auditory system for representing the axes of complex spectral representation, the somatosensory and motor homunculus in sensory and motor cortex, and the conformal like representation of visual space in the visual field. In each case the spatial axis encodes the functional axis representation.

**Figure 1:**
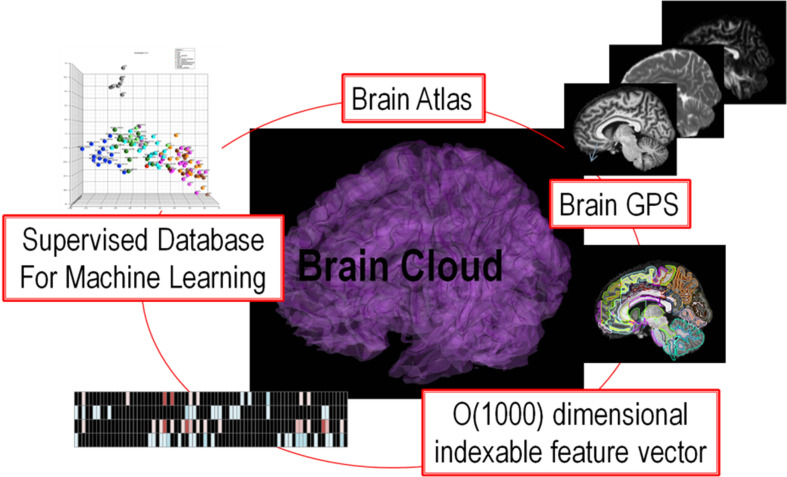
**Showing the core components of the high-throughput neuroinformatics pipeline including content (the atlas family), the personalization technology (GPS DiffeoMapping), and machine learning on the index or high-dimensional feature vector**.

The fact that functional topography is supported via dense topologic correspondence to the anatomical coordinates is the basis of our personalization of atlas based neuroinformatics. The personalization step is accomplished via the construction of a positioning system for neuroinformatics termed DiffeoMaps. This is an infinite dimensional positioning system which we term a Geodesic Positioning System (GPS) (Miller et al., 2013b) transferring information between atlas or world coordinate systems and individualized coordinate systems. We term it geodesic positioning since the metric is constructed based on the shortest (geodesic) flow of diffeomorphisms which connect the coordinates (Miller et al., 2013b). Such a transfer of the atlas representation to the coordinates of the individual allows for the organization of the high-throughput medical image record into a high-dimensional “feature vector” or an “index.” Indexing via DiffeoMaps is the essential reduction or parsing of the individual into metadata representations upon which the machine learning phase of high-throughput neuroinformatics may be applied. Shown in Figure 1 is our overall solution for high-throughput neuroinformatics, which includes atlases, diffeomorphic mapping for position (GPS), reduction to a high-dimensional feature vector or index encoding the anatomical and functional phenotypes, and machine learning via supervised clustering. This paper examines (i) cross-sectional image analysis to evaluate the health status of individual subjects with respect to the population data, (ii) integration of image and non-image information for diagnosis and prognosis.

### Related works

The high-throughput Neuro-Imaging Informatics introduced in this article is based on three core technologies; deformable multi-modal brain atlases, geodesic positioning of meta-data or semantic labels via diffeomorphic image transformation, and machine learning algorithms, as detailed in the Materials and Methods below. The deformable multi-modal brain atlases have been developed in both the coordinate systems provided by Montreal Neurological Institute (MNI) and the International Consortium of Brain Mapping (ICBM), which is a multicenter effort known for the MRI database and various brain atlases (http://loni.usc.edu/ICBM/). The atlases developed through this consortium have been implemented in leading software packages for the functional and anatomical brain analyses, such as Statistical Parametric Mapping (SPM, http://www.fil.ion.ucl.ac.uk/spm/), FSL (http://fsl.fmrib.ox.ac.uk/fsl/fslwiki/), MRICron (http://www.mccauslandcenter.sc.edu/mricro/mricron/), as well as our own MRIstudio (https://www.mristudio.org/). The primary uses of the MNI or ICBM atlases are to be a reference space for the voxel-based image analysis, in which statistical analyses are performed on each voxel after all images are normalized to the atlas space. This voxel-based approach has been widely used since it enables researchers to statistically analyze the whole brain with very high spatial specificity, and to report their findings on a standardized coordinate system. This approach also allows users to apply various types of anatomical parcellation maps, such as the automatic anatomical labeling atlas (AAL) (Tzourio-Mazoyer et al., 2002) and the LONI Probabilistic Brain Atlas (LPBA) (Shattuck et al., 2008) for quantifying gray matter functions and anatomy. Our deformable multi-modal brain atlases are extensions of these attempting to group voxels based on anatomical or functional units, through which features of each brain are preserved at 1 mm scale. While most of the “atlas-based” approaches previously have targeted the gray matter areas of single contrast images, the atlas used in our approach is multimodal, which means that the atlas consists of a set of images with different contrasts [e.g., T1- and T2-weighted images, Diffusion Weighted Imaging (DWI), Diffusion Tensor Imaging (DTI), and Susceptibility Weighted Image (SWI) contrasts] to allow multimodal image analysis of both gray and white matter structures in the common anatomical framework. The multimodal capability is supported by the Large Deformation Diffeomorphic Metric Mapping (LDDMM) methods that employ single and multi-channel algorithms (Beg et al., 2005; Ceritoglu, 2008; Ceritoglu et al., 2009; Djamanakova et al., 2013), allowing for the incorporation of multiple imaging modalities while performing simultaneous mapping that maximally satisfies registration of the multiple modalities.

Another distinction we have made is to explicitly model both the geometric component of the atlas and associate to that the anatomical phenotype, simultaneously with the contrast component of the atlas which we generally associate to the function. This we do by providing a direct model in which the anatomical geometry carries the function, and demonstrate explicitly how to code via informatics both the anatomical geometry simultaneously with the functional contrasts. This forms the heart of our personalization via DiffeoMaps below. This allows us to directly generate classifiers and perform hypothesis generation about disease groups by both the anatomical phenotype as well as the contrast or function phenotype, and index them to different atlases. This can be contrasted to alternative approaches which use normalization viewing the geometric or anatomical phenotype as a nuisance parameter which is normalized out, like the affine group is removed rather than explicitly modeled.

Since the robustness of the machine learning framework to detect disease related anatomical and functional features of the brain has been demonstrated (Teipel et al., 2007; Hinrichs et al., 2011; Zhang et al., 2011), our approach is the generalizable extension toward high-throughput whole-brain multimodality analysis of heterogeneous brain conditions.

## Materials and methods

### Atlas representation of 1 mm structural—functional contrasts

**Figure 2:**
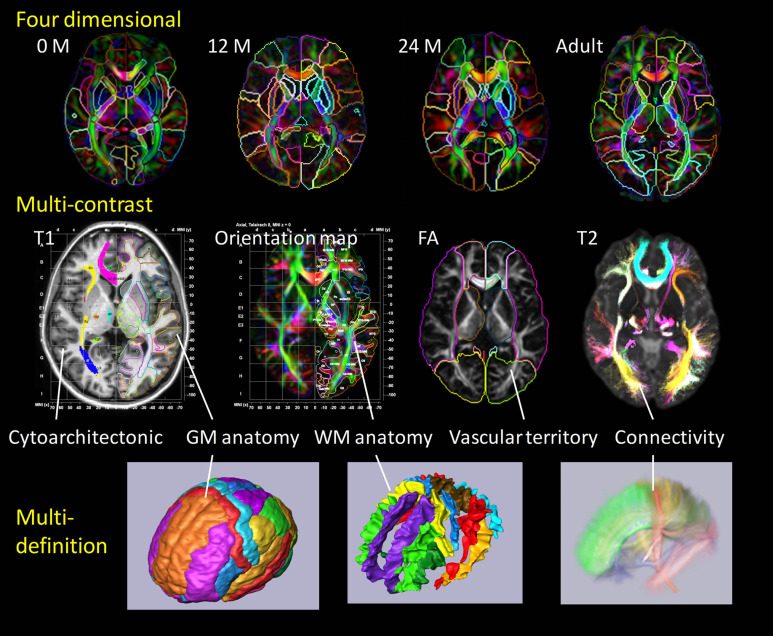
**Panels show a current brain atlas, including 3D anatomical information at different developmental stages (multi-dimension), different MR contrasts (multi-contrast), and different structural definitions**. The coordinate systems include MNI and Talairach coordinates with the brain depicted as parcellated into multiple cortical and subcortical areas including deep gray and white matter structures based on anatomical features (anatomical parcellation) as well as functional parcellation based on cytoarchitecture, vascular territories, and anatomical and functional connectivity.

The core of our high-throughput neuroinformatics technology is the conversion of the raw images into a structured, quantitative, and searchable high-dimensional feature vector. The basis for reduction to the numerical knowledge representation are the evolutionarily stable categorizations which neuroscientists have defined over the past decade. Our starting point is dense atlases of neuroanatomical structure and function indexed against age and group. We model the individual's imagery as an orbit under transformation of 1 mm scale coordinatized atlas information. Figure 2 depicts our coordinatized human atlases demonstrating 3D anatomical information at different developmental stages (multi-dimensional) (Oishi et al., 2011c), different MR contrasts (multi-contrast) (Oishi et al., 2009), and varying coordinatized structural and functional definitions (Mori et al., 2013). The coordinate systems support MNI (Mazziotta et al., 1995, 2001) and Talairach (Talairach and Tournoux, 1988) coordinates as well as parcellations into different cortical areas as well as approximately 20 deep gray matter and 100 deep white matter structures all based on anatomical parcellation. The cortical partition includes structures such as parietal gyrus, frontal gyrus, pre-central gyrus, cuneus, lingual and others; the subcortical structures include amygdala, caudate, globus pallidus, hippocampus, putamen, thalamus, red nucleus, substantia nigra, hypothalamus, nucleus accumbens; the white matter structures include corticospinal, internal capsule, thalamic radiation, corona radiate, fornix, longitudinal fasciculus, corpus callosum, and others. Such a modern atlas also includes parcellations based on different anatomical and functional criteria such as cytoarchitecture, vascular territories, and anatomical and functional connectivity. This type of effort to parcellate the brain has been a subject of research based on histology (von Economo and Koskinas, 1925; Sarkisov et al., 1955; Mai et al., 1997; Schleicher et al., 1999; Tzourio-Mazoyer et al., 2002; Zilles et al., 2002) or MRI for the cortex (Lancaster et al., 2000; Mazziotta et al., 2001; Tzourio-Mazoyer et al., 2002; Hammers et al., 2003; Maldjian et al., 2003; Shattuck et al., 2008), white matter (Meyer et al., 1999; Mori et al., 2008; Oishi et al., 2008) and the whole brain (Fischl et al., 2002; Desikan et al., 2006; Oishi et al., 2009, 2011c, 2013).

### Personalization via diffeomaps as a geodesic positioning system

Reduction to a high-dimensional feature vector which can be indexed requires us to model the high-throughput imagery. The underlying assumption of our model is that the meta-data representing the individual's structure and function is carried by the individual's coordinate systems, and there exists a structure preserving mapping which transforms the individual's coordinates into the stereotypical atlases. We term these transformations morphisms, these transformations form a group ϕ ∈ *G*. The structure preserving morphisms provide correspondence between “charts” of the human brain as contained within atlas and the individual's coordinates. In this sense the morphisms provide a positioning system through their algebraic group action. Our group has come to call this the *metamorphism* model (Miller and Younes, 2001; Trouvé and Younes, 2005), organizing the structural and functional informatics, the images *I* ∈ ℑ into the transformation –image pair [ϕ (*x*), *I*(*x*)], *x* ∈ *X* related via the algebraic pairing

(1) $$•:(\phi ,I)\mapsto I^{\prime }≐\phi •I\in ℑ.$$

In this model the morphisms denoted by ϕ(*x*), *x* ∈ *X* carries the coordinatized contrast metadata imagery denoted by *I*(*x*), *x* ∈ *X*.

Personalization occurs via smooth transformation of the atlas meta-data ϕ · *I*_atlas_. For this we define a distance inf_ϕ_*d*(*I*, ϕ · *I*_atlas_) between the individual's representation and transformed atlas solving a variational problem for the coordinate (Dupuis et al., 1998; Beg et al., 2005; Ceritoglu et al., 2009) transformation. The correspondence between the individual and atlas is termed the “DiffeoMap,” which provides an infinite dimensional positioning between atlas and world coordinates. This is in sharp contrast to the 7-dimensional similarity maps used in geographic positioning. To see this, the Eulerian velocities of Equation (2) below, while spatially smooth are a high-dimensional field, implying the Jacobian expressing first order transformation of coordinates in space allows the tissue to locally scale and twist while at the same time preserving relative organization.

**Figure 3:**
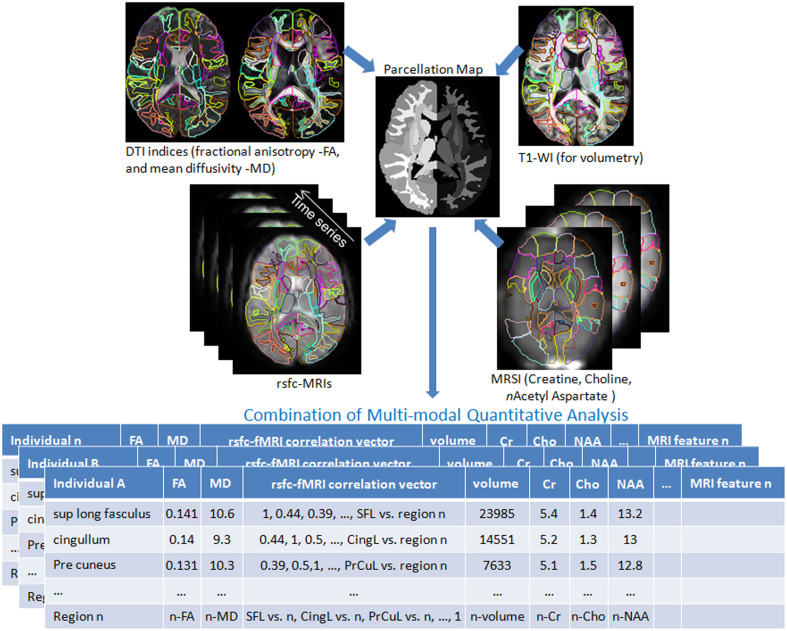
**Multiple image contrasts obtained from an individual using different MR pulse sequences**. These multiple images are simultaneously parcellated into multiple structures, linking their coordinate systems (Parcellation Map). This procedure reduces the vast anatomical information into a parcellated series of approximately 200 structures and a series of MR contrast values that are the signature of each individual. DTI: Diffusion Tensor Image, T1-WI: T1 weighted images, rsfc: resting state functional connectivity, MRSI: Magnetic Resonance Spectroscopy Images. This pipeline is available at http://www.mricloud.org/ (Tang et al., 2013b).

Shown in Figure 3 are instantiations of our structure-function metamorphosis model, including structural contrast imagery T1, orientation vector imagery such as DTI, metabolic contrast as measured via magnetic resonance spectroscopy and functional connectivity via resting-state fMRI (Faria et al., 2012).

Each of the modalities has its own definition of the morphism acting on the meta-data of the contrast imagery explicating the algebra represented by •, specifically (i) for the submanifolds of subcortical structures, gyral curves and cortical surfaces the morphism acts ϕ · *x*=ϕ (*x*), (ii) for scalar imagery such as T1 the morphism acts via the inverse ϕ · *I* = *I* ◦ ϕ^−1^, and (iii) for symmetric matrix-valued DTI (color in Figure 1) with eigen elements {λ_*i*_, ϕ_*i*_}, the morphism acts to preserve the eigenvalues and determinant, rotating the eigenvectors $\phi \cdot I\dot{=}\left(\lambda_{1}\underset{1}{\hat{e}}\overset{t}{\underset{1}{\hat{e}}}+\lambda_{2}\underset{2}{\hat{e}}\overset{t}{\underset{2}{\hat{e}}}+\lambda_{3}\underset{3}{\hat{e}}\overset{t}{\underset{3}{\hat{e}}}\right){}^{\circ}\phi^{-1}$ with ${\hat{e}}_{1}=\frac{(d\phi ){\hat{e}}_{1}}{\left\|(d\phi ){\hat{e}}_{1}\right\|}$, ${\hat{e}}_{2}=\frac{(d\phi ){\hat{e}}_{2}-\langle {\hat{e}}_{1},(d\phi ){\hat{e}}_{2}\rangle {\hat{e}}_{1}}{\sqrt{{\left\|(d\phi )e_{2}\right\|}^{2}-{\langle {\hat{e}}_{1},(d\phi ){\hat{e}}_{2}\rangle }^{2}}}$, ${\hat{e}}_{3}={\hat{e}}_{1}\times {\hat{e}}_{2}$ and $d\phi =\left(\frac{\partial \phi_{i}}{\partial x_{j}}\right)$ the 3 by 3 Jacobian matrix, with *x* denoting the vector cross-product.

### The high-dimensional feature vector and machine learning

The scanners are the high-throughput devices generating the high-dimensional raw images of O(10,000,000) in complexity, and the pipeline converts it into a quantitative searchable feature vector {*f* = *X*_1_, *X*_2_, *X*_3_, …} representing the individual at O(1000–10,000) complexity. Diffeomorphic GPS (Miller et al., 2013b) provides the basis for data reduction, since the anatomical structure phenotype is encoded by the morphisms and the meta-data of structure-function are encoded by the contrast imagery represented in atlas coordinates. The metamorphism model organizes the structural and functional informatics into the pair [ϕ(*x*), *I*(*x*)], *x* ∈ *X*.

The GPS correspondences are diffeomorphisms, one-to-one and smooth mappings between coordinate systems ϕ : *X* ↔ *Y*, ϕ (*x*), *x* ∈ *X* providing correspondences ϕ: *I*↔ *I*_atlas_ between the individuals in the population and the atlas. The correspondences are generated as solutions of the classical Lagrangian flow equations, ${\dot{\phi }}_{t}=v_{t}(\phi_{t}),t\in [0,1]$ the time derivative of the flow *v*_t_ is termed the Eulerian velocity (Christensen et al., 1996). Constructing the DiffeoMaps occurs via the geodesic connection of one coordinate system to the other (Miller et al., 2006), solving for the geodesic connection between individual *I* and atlas ϕ · *I*_atlas_ according to

(2) $$\inf_{v_{t,t\in [0,1]}:\dot{\phi }=v(\phi ),\phi_{0}\cdot I_{\text{atlas}}=I_{\text{atlas}}}\int_{0}^{1}{\left\|v_{t}\right\|}_{V}dt\text{ subject to }I=\phi_{1}\cdot I_{\text{atlas}}.$$

The geodesic connections are encoded via their initial tangent vector at the identity, denoted as ν = *v*_*t* = 0_ ∈ *V*. This forms the natural coordinate system of our GPS (Miller et al., 2013b). We have reduced the anatomical phenotype to a set of coordinates ν = *v*_*t* = 0_ ∈ *V* centered at the atlas.

This is a natural representation of the anatomical or shape phenotype since the norm of the coordinates preserves the metric structure on the space of anatomies using this framework (Miller et al., 2006). The shortest flows connecting the template and individual coordinate systems define the metric in this space, the metric of Equation (2) is given by the integrated norm of the vector fields generating the morphisms. The reduction of the shape phenotype to these diffeomorphic connections we call *diffeomorphometry* (Miller et al., 2013b). At the 1 mm scale of MR imagery the anatomical phenotype is extremely sparse relative to the high-dimension of the initial data. For smooth imagery such as MRI linear functions of the vector fields, termed the shape momentum, are concentrated to the boundaries of the homogeneous subcomponents of the object (Miller et al., 2006; Qiu and Miller, 2008). At places in the image that are constant the shape is coded as zero. Plainly put, at the 1 mm scale gyral and subcortical regions of the MRI contrasts do not discriminate the cellular architecture.

**Figure 4:**
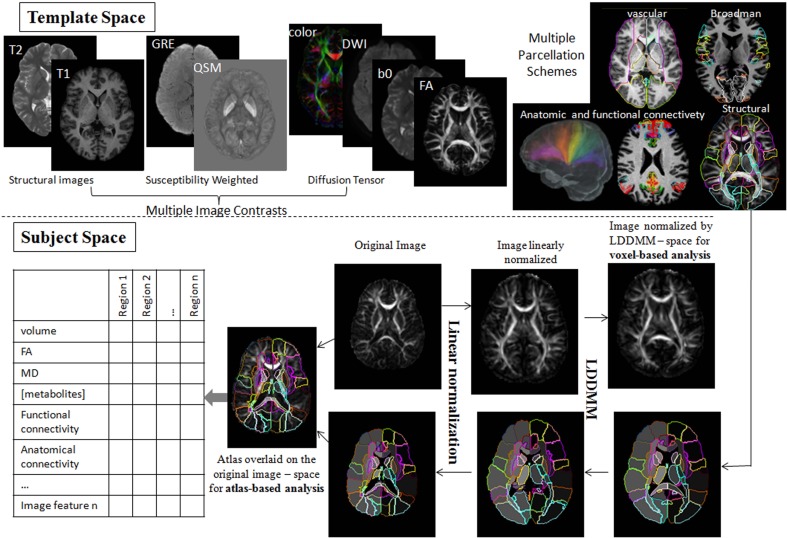
**Showing the pipeline starting with the modalities in atlas coordinates (top row) with the DiffeoMap applied to the target showing the parcellation of target modalities (bottom row)**. The algorithm used for solving for the multi-modality DiffeoMap is multi-modality LDDMM.

Shown in Figure 4 is an instantiation of our pipeline, depicting the personalization phase via DiffeoMap. The generation of the geodesic of Equation (2) for image matching via the solution of a quadratic variation problem on the vector field we call large deformation diffeomorphic metric mapping (LDDMM) (Beg et al., 2005). The modalities are shown in the top row in atlas coordinates with the DiffeoMap applied to the target showing the parcellation of target modalities shown in the bottom row.

**Figure 5:**
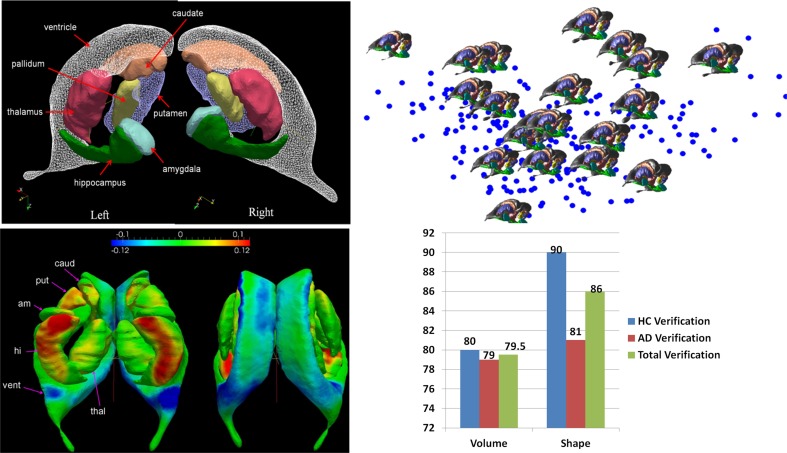
**Top row:** Panel shows the left-right subcortical structures for human at 1 mm including amygdala (A, light blue), caudate (C), hippocampus (H, green), globus pallidus (PAL), putamen (PUT), ventricle (VL), thalamus (TH). The right panels shows the layout in the geodesic coordinate system of 250 of the anatomies (blue dots) in the first two dimensions with 20 of the brains shown explicitly with the basis dimensions on the order of 20–40 dimensions for each subcortical structure occupying 95% of the variatiance of anatomical variation with ordering A20<PAL22<C25<PUT27,VL27<H30<THA40. **Bottom row**: Shows the geodesic coordinates of the population (top right) relative to the atlas (top left) shown as a shape statistic computed by averaging over all geodesic mappings and computing the Jacobian of the tangent vector at the identity representing the anatomy. Bottom left shows the difference in the means μ^*HC*^ − μ^*AD*^ superimposed on the template. The log-determinant of the Jacobian is shown, with red corresponding to shrinking and blue expansion. The bottom left panel depicts the hippocampus and amygdala are significantly red means large shrinkage relative to the contols, with the blue signaling the expansion of the ventricles. Bottom right panel shows a classifier based on three structures using only volume (left hand) and all the 20–40 dimensions of the anatomical phenotype encoded by the geodesic coordinates for hippocampus, amygdala, and ventricle. The images used for this analysis are a portion of a dataset published with the methodological detail (Tang et al., 2013a).

Figure 5 shows a depiction of the subcortical neuronanatomy atlas as measured in 1 mm scale MR. The left panel shows the atlas of 14 subcortical structures, amygdala (A, light blue), caudate (C), hippocampus (H, green), globus pallidus (PAL), putamen (PUT), ventricle (VL), thalamus (TH), each surface in the atlas containing order 1000 vertices. The set of structures correspond to an atlas generated from the population of healthy controls (HC) and Alzheimer's disease (AD) computed using the surface template estimation algorithm described in Ma et al. (2010). To demonstrate the sparcity of the anatomical phenotype at 1 mm scale, the geodesic correspondence between the atlas and a database of 250 subcortical brains were generated giving a coordinate identification of each element in the population, *I* ~ *v*, where *v* is the geodesic coordinate representation of the anatomy to the atlas. To understand the variation over the population, they were expanded via principle component analysis into a basis $v(f_{1},f_{2}\ldots )=\sum_{i}f_{i}U_{i}$; the *f*'s are reduction of the anatomical phenotypes to the basis of eigenfunctions *U*. The sparsity of the anatomical phenotype was calculated across the population calculating the dimension required for encompassing 95% of the energy of subcortical variation. Generally each structure requires between 20 and 40 dimensions, with hippocampus and thalamus having the greatest shape variation within the population in terms of number of dimensions. The 95% variance cutoff as a function of dimensions for each structure is A20<PAL22<C25<PUT27,VL27<H30<THA40, the sparse subcortical shape phenotype at 1 mm scale is O(1000). The right panel shows the layout in the geodesic coordinate system of 250 of the anatomies (blue dots) in the first two geodesic dimensions with 20 of the brains shown explicitly.

This huge data reduction is noteworthy as it is the direct generalization of the sparsity of rigid body momentum which itself encodes translation and angular momentum to single 3-vectors, even though the inertia is extended over the entire object. Taking the midbrain as roughly 1/3 of the total brain volume of 2–4 Million voxels implies a data reduction of three orders of magnitude to O(1000).

### Cortical, subcortical and white matter parcellation feature vector

The global positioning solution provides registered coordinates for the encoding of the target coordinates system into a parcellation corresponding to the anatomically defined partition of atlas coordinates in the 200 white and gray matter parcels. Denoting the atlas partition *p*_*i*_, and since there can be as many as 7 MR contrast values including T1, T2, B0, trace, FA, spectroscopy, gives O(1000) features

(3) $$f_{P_{i}}^{c}=\int_{P_{i}}I^{c}(x)dx,i=1,\ldots ,200,c=1,..,7.$$

**Figure 6:**
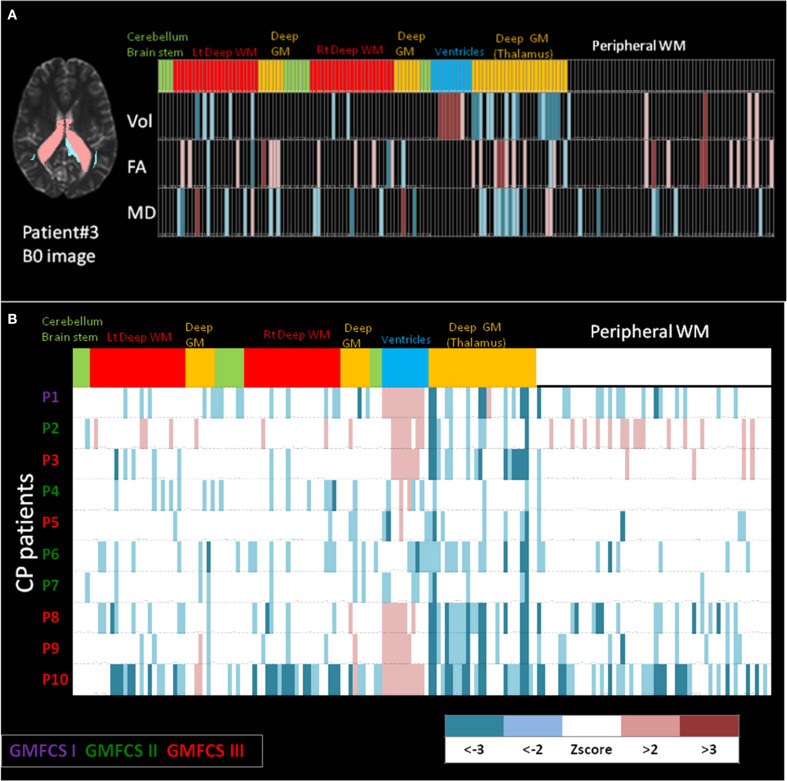
**(A)** Feature vector from the personalization DiffeoMap correspondence between the atlas and an individual's coordinate system associated with focal disease category. Informatics partition with 200 structures including, volume, FA and MD values into peripheral white and gray matter, and deep white and gray matter structures. The features are color coded according to the statistics to depict color-coded outliers: WM: white matter, GM: gray matter. **(B)** Example of population data including 10 cerebral palsy patients with different prognoses in their motor disability. Informatics partition with 200 structures of volume for each patient is shown as 10 rows. The features are color coded according to *z* scores calculated based on normal control population. CP: cerebral palsy, GMFCS: gross motor function classification system.

Shown in Figure 6 are examples of the neuroinformatics parcellation which is transported via the personalization phase. Figure 6A shows the DiffeoMap personalization of the atlas into the coordinates of a spastic cerebral palsy patient with visually appreciable anatomical abnormalities (the color highlights the volume change larger than two standard deviations). The three rows show measurement results for volume, FA, and MD. Each column is an entry for one of the 200 anatomical structures. The top row represents anatomical information of each parcellated structure. In feature space, the neuro-informatics atlas supports both empirical means as well as empirical variances. Only features which demonstrate as outliers are depicted.

The bottom part, Figure 6B, shows an example of population data, in which the atlas partition of the anatomical phenotype for the listed structures (the bottom row in Figure 6A) are presented for 10 cerebral palsy patients (P3 is the individual shown in Figure 6A). All patients shared the same spastic phenotype with varying degree of motor impairment indicated by GMFCS scores. Abnormal parcellation volume values are presented by *z*-scores. At a glance, even though the patients were selected by similar clinical manifestations, a marked degree of anatomical variability can be recognized implying the importance of clustering on the spectrum of anatomical phenotype.

### Functional MRI and connectivity maps in atlas coordinates

**Figure 7:**
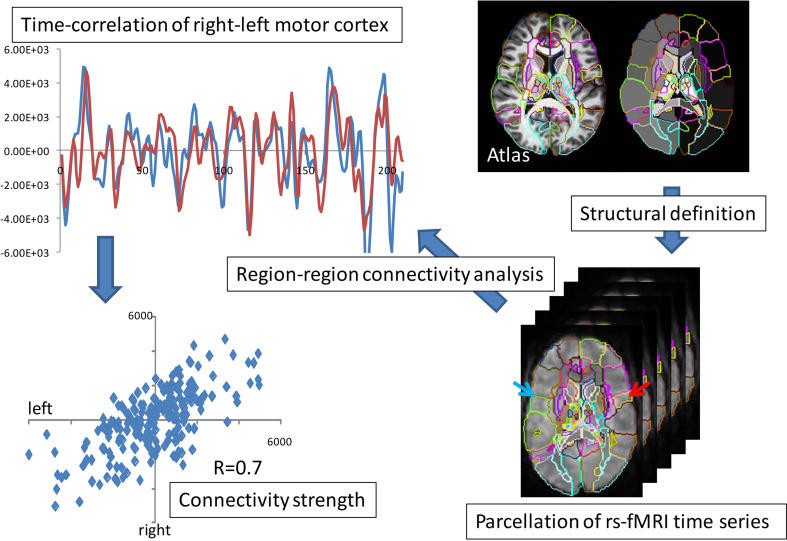
**fMRI parcellation based on resting state correlations**. The **top panel** shows the overlap of the resting state functional signals integrated over the right-left motor parcellation; the **bottom panel** shows the value of blue and red time series over the 210 time points.

Functional magnetic resonance imagery (fMRI) also provides ideal measurements for studying pairs of interactions in the brains. fMRI connectivity is based on empirical correlations of temporal responses between pairs of elements in the representation. Figure 7 shows an example of empirical correlation of fMRI at lag-0 using the common atlas coordinate system to parcelate symmetrically associated motor cortex areas plotting the resting-state MRI functions (rs-fMRI) (Tzourio-Mazoyer et al., 2002; Eickhoff et al., 2005; Achard et al., 2006; Hagmann et al., 2008; He et al., 2009; Wang et al., 2009).

Shown is the time series of the fMRI image modality *I*^fMRI^(*x*, *t*) integrated over the right and left motor cortex parcels. Notice the strong correlation depicted via the superposition of the red-blue time sequences. These highly correlated patches of tissue has resulted in the widely used ICA model in which the measured functional signal is the superposition of “networks,”

(4) $$I^{\text{fMRI}}(x,t)=\sum_{i}f_{i}^{\left(t\right)}U_{i}^{\left(x\right)}$$

the *U*'s playing the role of the resting-state networks. Working in the registered coordinates of the atlas allows for the construction of these resting state networks in the parcellations of the atlas by simply replacing the functional MR signal by it's parcellated representation $I_{Pi}(t)=\int_{P_{i}}I_{\text{fMRI}}(x,\;t)dx,P_{i}=1,\ldots 200.$. The *f*'s are the dimensions of the functional MRI signal representing 10–20 resting state dimensions added to the feature vector.

### Machine learning investigation of disease-specific phenotypes

High spatial resolution is one of the most significant advantages of clinical MRI and its usefulness in studying pathological condition and detecting abnormalities. It seems clear from many studies that because of the noise versus signal tradeoff most detectable pathologies from MRI are signaled via small groups of spatially correlated voxel contrasts. Dimensionality reduction becomes the central methodology for MRI analysis in clinical applications. Combining unsupervised principal component analysis (PCA) along with supervised training, on the supervised group means under the common covariance model gives linear discriminant analysis (LDA).

Given *m*-length feature vectors, a collection n of them {*f*_*j*_}, then PCA calculates the singular value decomposition (SVD) of the *m*× *n* matrix *F*= (*f*_1_, *f*_2_,…, *f*_*n*_ = *U*Σ*v*^*t*^, where *U* is an *m* × *m* orthonormal matrix of vectors with ∑ diagonal with entries the singular values. The connection to least-squares and covariance modeling of Gaussian processes is that the left singular vectors *U*= (*U*_1_,…, *U*_*m*_) are the eigenfunctions of the empirical covariance *FF*^*t*^; the set of diagonal entries squared of ∑ are the variances in the rotated independent representation of the left singular vectors. LDA then is the supervised version. Given groups of labeled feature vectors {*f*^*g*^_*j*_}, *g* = 1,… then each labeled group has a mean and covariance:

(5) $$\mu^{g}=\sum_{j=1}^{n_{g}}f_{j}^{g}/n_{g},K^{g}=\sum_{j=1}^{n_{g}}(f_{j}^{g}-\mu^{g}){\left(f_{j}^{g}-\mu^{g}\right)}^{t}/n_{g}.$$

Then LDA is PCA on the group means μ^*g*^ using the common covariance *K* = ∑_*g*_*K*^*g*^. Quadratic discriminant analysis is a particular non-linear discriminant analysis (QDA) relaxing the common across groups covariance assumption. The high-dimensional structural and functional phenotypes are encoded via high-dimensional feature vectors. The classifiers are constructed from the cohorts of neuropsychiatric illnesses collected via the supervised training component. The crucial advantage of this approach is that the anatomical and structural phenotypes are indexed to the coordinates of the template. For the subcortical structures, for example, the anatomical phenotype is immediately reduced from a feature vector of dimension O(10,000,000), to the dimension of the surfaces which is O(10,000). Similarly, the functional feature is indexed over the anatomical substructures. This of course requires the notion of a template coordinate system which is centered in the population. Unlike other methods since we have explicitly modeled the anatomical and functional phenotypes, we can perform classification on both rather than viewing coordinate system transformation as a nuisance variable.

## Results

### The anatomical phenotype: image retrieval and clustering

With an estimated 100 million scans every year in radiology, a huge amount of imaging data are generated every day, with these data stored in clinical Picture Archiving and Communication Systems (PACS) and are rarely used to support medical decision-making in cross-sectional examination of patient populations. Similar to the role of genomic and proteomic information for personalized medicine, anatomical phenotypes are fundamentally important for medical decision-making, yet often not systematically utilized in daily medical practice. While text-based patient records for retrieval of disease cohorts is commonly used, to utilize the anatomical phenotype for medical decision-making for individual patients we need to be able to use the patient image as the search key with the diagnostic label being the retrieved information. Using the high-dimensional feature vector without any diagnostic supervised labeling allows us to group and retrieve based on the structural phenotype.

**Figure 8:**
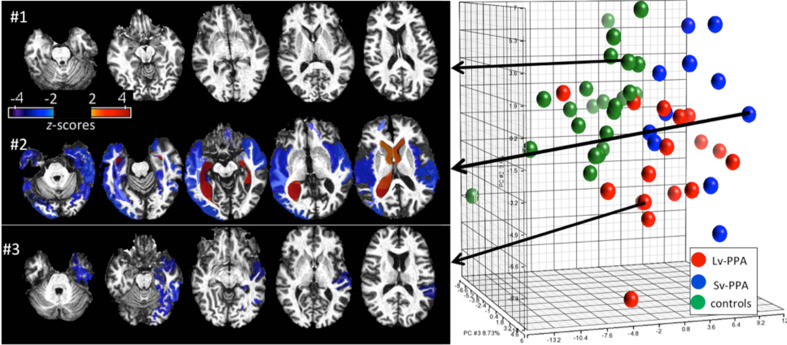
**Representation of degrees of regional atrophy as *z*-scores to support diagnosis (left panel)**. In cross-sectional studies on patients with similar diagnostic criteria, the patterns of atrophy from populations can be integrated with clinical information providing diagnostic and prognostic information. Clustering on the dimensions of the anatomical phenotype (**right panel**). The PCA plot contains the volumes of 200 brain structures in 24 healthy controls and 28 PPA patients. Shown are groupings according to the anatomical features associated to the clinical labels (controls, SvPPA, LvPPA).

Figure 8 shows an example of retrieval based on the anatomical phenotype, essentially delivering previously supervised cases with clinical information already in the data base. There are two types of information delivered in this analysis. For this we represent the anatomical variance of the population as shown in Figure 5 for the subcortical structures to represent the coordinates of the anatomical position of the patient with respect to the atlas coordinate system relative to the population. This can be highly illustrative. For example, Figure 8 shows healthy individuals as controls (green dots in the PCA plot of the structural volumes) and patients with two variants of Primary Progressive Aphasis (PPA), a neurodegenerative disease characterized by predominant and progressive deterioration in language in the absence of major change in personality, behavior or cognition other than praxis for at least two years. The *z*-score map in patient #3 reveals atrophy at the temporal left side that could be dubious at visual inspection only. In the addition, this subject is closer to other PPA patients than to the controls in the PCA plot, evidencing that the anatomical phenotype identified agrees with the clinical label.

Defining cohorts of similar patients is commonly done based on a host of features, including clinical behavioral and structural and functional phenotypes as measured in the functional and structural imagery. Figure 8 examines clusters of cohorts based solely on the anatomical phenotype feature vector. The first principal component (PC1) accounts for global cortico-subcortical atrophy and ventricle enlargement, and mainly segregates age-matched controls from the PPA population. The segregation between two PPA variants (Semantic-SvPPA and Logopenic-LvPPA) is driven by severe and global atrophy of deep areas in SvPPA (PC2) and the predominant fronto-parietal atrophy in LvPPA, with relative preservation of temporal areas, particularly left, when compared with SvPPA (PC3). This agrees with past anatomical qualitative description of these populations. The existence of “outliers” corresponding to patients or controls surrounded by subjects of different labels are due to the singular anatomical features of these subjects. This type of analysis provides a platform for hypothesis-free comprehensive characterization of anatomical phenotype. Such quantitative analysis allows the investigation of various anatomy-associating factors, such as disease progression and functional outcomes, in a systematic manner.

### Disease quantification of anatomical phenotype via geoodesic coordinates

The GPS provides geodesic coordinates for representing every element in the population relative to the templates. We have examined machine learning on the subcortical structure coordinates shown in Figure 5. In the ADNI (Mueller et al., 2005) project there is extensive diagnostic supervised labeling enabling group based discriminations such as LDA/QDA for cross-sectional study of cohorts in dementia. A total of 385 subjects were segmented into their subcortical structures and lateral ventricle using FreeSurfer (Fischl et al., 2002) based on the analyses published by the Dale group (Fennema-Notestine et al., 2009). There were a total of 210 HC and 175 subjects with AD. To illustrate the average differences between the healthy control and Alzheimer's disease populations a HC-template surface and an AD-template surface was generated representing the center of each of the populations. We compared these two, HC-only and AD-only, template surfaces from the two different populations, and which are represented in geodesic coordinates relative to the overal template representing both HA-AD populations via the two class means μ^*HC*^, μ^*AD*^. To visualize these as shapes we calculated a scalar field corresponding to the log-determinant of the Jacobian of the map between the two averages, and visualized it on the template surface generated from the HC population generated using the algorithm described in Ma et al. (2008, 2010). This scalar field measures how much expansion/atrophy at each vertex of averaged surface from AD compared to that from HC in the logarithmic scale: i.e., positive value corresponds to surface expansion in the AD averaged surface at a particular location, while negative value denotes surface atrophying. The bottom left panel of Figure 5 shows the mean differences between the two populations (bottom left), and is a visualization of one of the direction vectors in the Fischer discriminant the difference between the means μ^*HC*^ − μ^*AD*^, shown as a plot of the Jacobian determinant. The color red represents the determinant being less then one, corresponding to shrinkage. The blue color corresponds to expansion. We see most of the shape change occurring at the ventricle expansion and the hippocampus and amygdala shrinkage.

The bottom right panel of Figure 5 shows the result of building classifiers via machine learning whose discriminating dimensions are encoded in the picture in the lower left panel. We constructed the LDA and quadratic QDA classifiers using one of the *Leave-One-Out Cross-Validation* resampling method generating 385 LDA classifiers, testing on one of the subjects treating them as the testing data, and constructing the LDA class means μ^*HC*^, μ^*AD*^ from the other 384 subjects (Tang et al., 2013a). For the two class problem the discriminating direction resulting from LDA on the geodesic coordinates is the projection of the differences in the means according to *K*^−1^(μ^*HC*^− μ^*AD*^) on the common covariance. The Bayes classifier for the two class problem becomes a comparison to a threshold of the inner product of the feature vector on the discrimating direction:

(6) $$f^{t}K^{-1}{\left(\mu^{HC}-\mu^{AD}\right)}_{AD<}^{HC\geq }\theta .$$

As shown in Figure 5 we find uniformly, as depicted by the blue bars in the classifier diagram, that the shape dimensions associated with the subcortical structures are significantly more discriminating then the volumes, generally reducing the errors in discrimination by more than 10%. The significant dimensions in volume and shape are associated with hippocampus and amygdala agreeing with previous results (Qiu et al., 2009). The specificity and sensitivity based on using the PCA shape dimensions in the feature vector for these three subcortical structure phenotypes is 90 and 81%, respectively. This is consistent with recent findings in another preclinical dementia study (Miller et al., 2013a) in which the shape of the temporal lobe subcortical structures is more discriminating then volume measures as well as in a Huntingdon's disease study tracking caudate, putamen, and globus pallidus (Younes et al., 2012).

### Personalized analyses: predicting future conversion based on white matter structural representations

While most research studies are based on cross-sectional population-based analyses, clinical diagnosis is always based on single individuals. This is performed by visual inspection in daily radiological diagnosis, in which images are most likely analyzed in a structure-by-structure basis, not in a voxel-by-voxel basis. Atlas-based neuroinformatic analyses in terms of their aggregate scale of the feature vector is compatible with many current diagnostic practices. Interestingly, histopathological studies indicate that white matter is an excellent target for both the early diagnosis of AD and for monitoring disease progression, motivating the use of DTI for studying patients with AD (Brun and Englund, 1986; Englund et al., 1988; Meier-Ruge et al., 1992; Gunawardena and Goldstein, 2001; Pigino et al., 2003; Sjobeck et al., 2005; Stokin et al., 2005; Chevalier-larsen and Holzbaur, 2006; Oishi et al., 2011b). There are already a large number of cross-sectional group comparison studies reporting significant differences in DTI derived measurements between the patients and controls, suggesting that white matter damage may exist in the pre-symptomatic phase of AD (Rose et al., 2000; Kantarci et al., 2001; Medina et al., 2006; Ringman et al., 2007; Stahl et al., 2007; Zhou et al., 2008; Damoiseaux et al., 2009; Salat et al., 2010; Sexton et al., 2011). One of the important questions after group analyses is whether these findings can be applicable to each individual to predict future conversion from memory impairment without other cognitive deficits (amnestic mild cognitive impairment) to dementia caused by AD. This is important because the amnestic mild cognitive impairment is a clinical category including multiple diseases or conditions with different pathological background, and not all of them develop AD (Albert et al., 2011).

**Figure 9:**
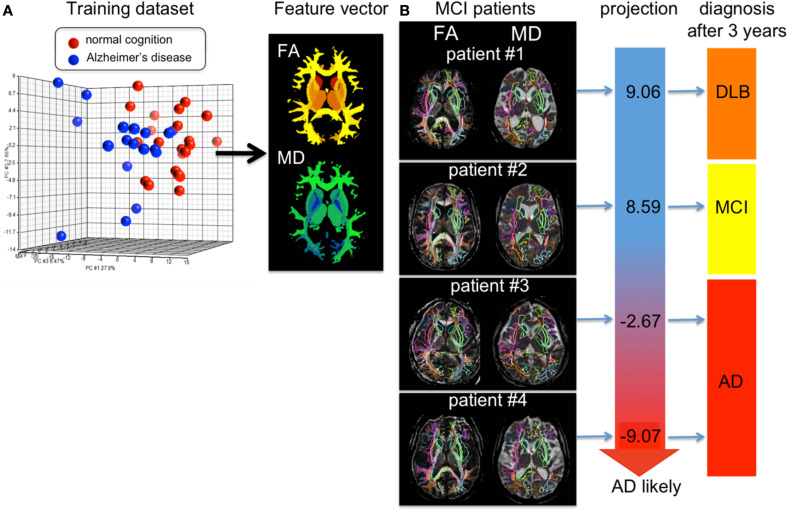
**(A)** shows the result of PCA of the DTI derived measurements (FA = fractional anisotropy and MD = mean diffusivity) from 136 white matter areas and 12 deep gray matter structures. The first component was used as a diagnostic feature vector; the brighter area indicates more weighting to a degree of FA reduction and the cold brighter area indicate more weighting to a degree of MD increase to separate the AD group from the control group. **(B)** For individual images, the atlas was DiffeoMapped and projection to the feature vector was calculated. The projection well predicted early conversion from amnestic mild cognitive impairment (MCI) to Alzheimer's disease (AD), but did not predict conversion from MCI to the dementia with Lewy body (DLB). The DTIs used for this analysis are a portion of a dataset published in Oishi et al. (2011a).

Figure 9 shows results of personalizing the cross-sectional atlas statistics to several patients. A weighted feature vector, which could separate AD from cognitively normal population, was created from training datasets including groups of patients and cognitively normal age-matched individuals using dimensionality reduction applied to the atlas feature vector, and then DiffeoMapped to each individual to calculate the projection onto each patient. Shown in Figure 9 are examples of the prediction of the conversion to Alzheimer's dementia in successive followup. Notice this projection doesn't predict conversion from amnestic mild cognitive impairment to the dementia with Lewy body, which is another type of neurodegenerative dementia. This type of analysis requires a large database with longitudinal follow up which is an important current focus of our efforts.

### Discriminating between multiple diseases

**Figure 10:**
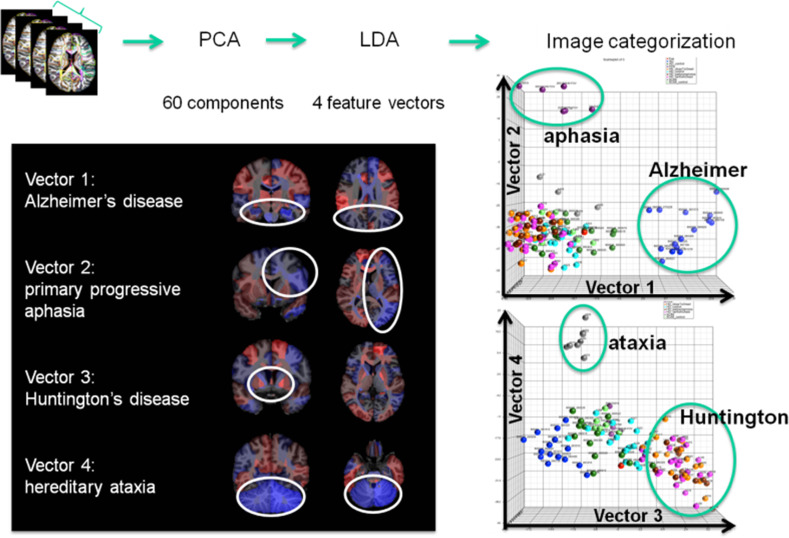
**Showing four clinically labeled disease categories of Alzheimer's Disease, aphasia, Huntington's, hereditary ataxia, and one control group upon which the anatomical features were learned including 60 PCA dimensions followed by supervised LDA delivering 4 loading vectors for discrimination**. The clustering features in the high-dimensional index on the **right** are shown to correspond to anatomically meaningful shape representation shown in the **left panel**.

The concept of group analysis in research studies assumes consistent locations of abnormalities, which does not hold for clinical situations, with heterogeneous patient populations and lack of an age-matched control group. The atlas-based neuroinformatics is compatible with the analysis of multiple diseases with different anatomical features. Figure 10 shows the applicability of atlas-based neuroinformatics to capture anatomical features of multiple neurodegenerative diseases with known macroscopic anatomical alterations. To appropriately integrate diagnostic information to characterize the anatomical features related to each disease category, PCA and LDA were applied sequentially to a dataset consisting of 102 T1-weighted images from AD, primary progressive aphasia, Huntington's disease, hereditary spinocerebellar ataxia and normal control participants. These were parcellated based on the JHU-atlas [the images used for this analysis are a portion of a dataset published with the methodological detail (Qin et al., 2013)]. The weighted feature vectors efficiently captured known disease-specific anatomical alterations. For example, the medial temporal lobe and the parietal lobe were negatively weighted in the feature vector of AD to give a higher discriminant score for AD compared with other diseases and the control group. It should be noted that ventricular enlargement was not emphasized in the feature vector, although it was seen in most of the AD patients. Ventricular enlargement has been regarded as one of the disease-related features in past studies based on a cross-sectional comparison between AD and a control group, but seems to contain less information for separating AD from other neurodegenerative diseases.

### Functional MRI phenotypes in atlas coordinates

**Figure 11:**
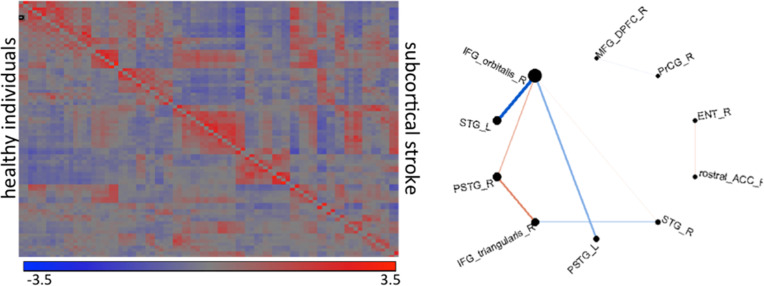
**The intensity plot shows average of Fisher-transformed 84 × 84 correlations of rs-fMRI response in atlas partition in individuals with subcortical stroke (superior to the diagonal) and controls (inferior to the diagonal)**. The diagram shows the connections that are different between groups (*p* < 0.001); the thickness of the lines is proportional to the ratio of the correlations (stroke/controls); blue are correlations with opposite signal between groups (positive—negative); red are those with same signal. R: right hemisphere, L: left hemisphere, IFG_orbitalis and IFG_triangularis: pars orbitalis and triangularis of the inferior frontal gyrus, MFG_DPFC: dorsal prefrontal pars of middle frontal cortex, PSTG and STG: posterior and medial pars of the superior temporal gyrus, rostral_ACC: rostral pars of the anterior cingulate gyrus, PrCG: pre-central gyrus, Ent: entorhinal area.

Shown in Figure 11 are results from functional magnetic resonance imaging done in registered atlas coordinates. Given the accompanying structural T1 images the functional responses can be examined in atlas coordinates with hypotheses formed at the scale of the partition of the atlas. Shown is a comparison between 7 patients with stroke at deep gray matter (cortex is preserved) and age-paired HC. The intensity plot shows the average of Fisher-transformed correlations between the rs- fMRI time courses of each pair of 42 cortical regions in controls (bottom) and individuals with stroke (top). In general, correlations between temporal and frontal areas are the main source of differences between the groups.

### Clinical informatics and behavior phenotypes and functional phenotypes

**Figure 12:**
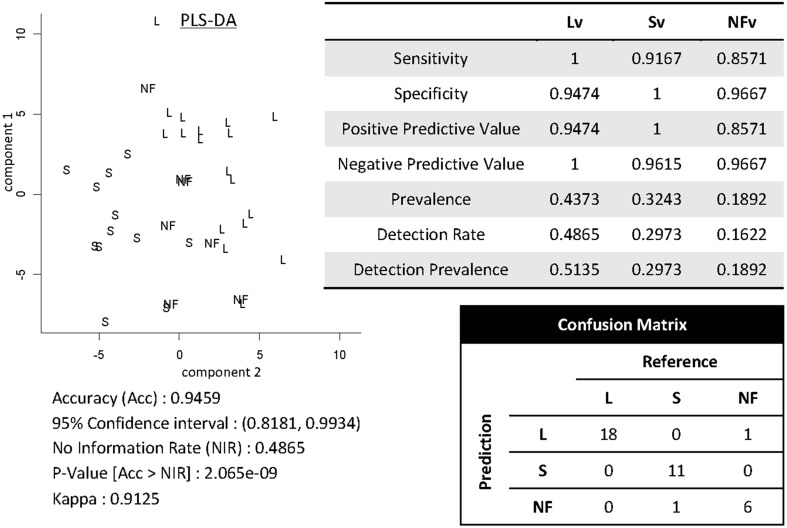
**Showing partial least squares—discriminant analysis (PLS-DA) for classifying 37 individuals into PPA variants based on volumetric data of 120 parcellated areas**.

Shown in Figure 12 are results of demonstrating high-throughput informatics used to classify individuals into clinical phenotypes based on functional MRI coupled to clinical behaviors. The 3 clinical variants of PPA (logopenic—Lv, semantic—Sv, and non-fluent—NFv) may differ in terms of disease progression and response to therapeutics. In the early stages of the disease, when some therapeutics are being tested and will hopefully be effective, the clinical tests are not always able to classify all the patients. In addition, although anatomical differences among these variants are reported at group level, the individual classification based on qualitative evaluation is not usually possible. High-throughput imaging informatics can contribute for individual classification. Figure 12 contains the volumetric data of 120 parcellated areas from 37 PPA patients that were scanned when, in their majority, the variant diagnosis wasn't completely clear, based on clinical information only. Our classification model, created using partial least squares—discriminant analysis (PLS-DA) and volumetric features (120 areas) demonstrated reasonable accuracy on predicting the variant diagnosis with a significant (higher than “by-chance”) *p*-value, both when tested by bootstrapping or by external testing sample. The detection prevalence is low, particularly in the smallest group (NFv) with the sample size needed to be increased.

High-throughput informatics is also an effective tool to scrutinize anatomical-functional/ behavioral correlations. Much of the mapping of brain functions has been via lesion based studies, by relating regions affected by a stroke or trauma, for example, with the functional deficit. Lesion-based studies, however, have significant limitations such as (i) areas most strongly associated with the deficit depend on the vulnerability to ischemia/trauma (ii) determining the part of the lesion which is responsible for the deficit is difficult, or whether it represents a reorganization of cognitive networks that are less efficient, and (iii) the challenge of determining the proportion of changes leading to functional recovery, more than functional loss, (iv) the lack of multiple parameters, local or widespread, that might be concomitantly affected and whose interaction might correlate with the deficit.

**Figure 13:**
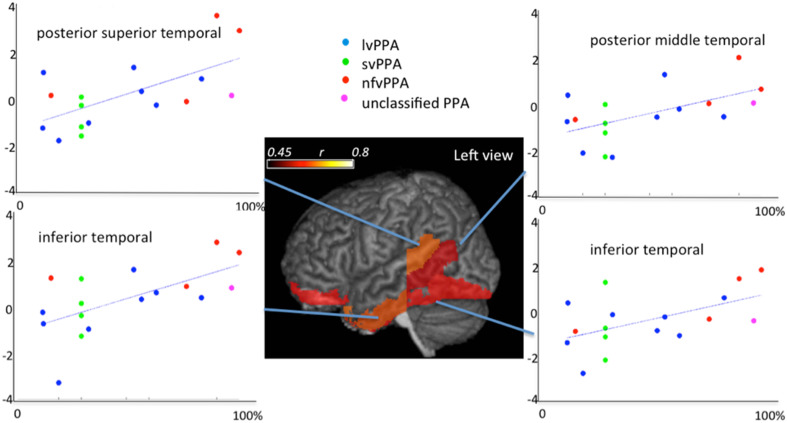
**Correlations between regional atlas anatomy (*z*-scores of volumes in *y* axis) and behavior (scores at Boston Naming Test—% of correctness in *x* axis) in individuals with Primary Progressive Aphasia—PPA**. Regions with significant correlations are colored and the color scale represents the degree of correlation. These data includes part of the dataset used in Race et al. (2013).

Shown in the Figure 13 is the application of quantitative analysis to assess anatomical-functional correlations in progressive disease models that affect specific functions (such as PPA, that affects primarily language) carried out by investigating the pattern of errors and their relationship to cortical impairment. It shows correlations between regional volumes and PPA patients' performance in a Naming test (Race et al., 2013). This type of anatomical-behavioral analysis provides a better understanding of the relationship between cognitive processes and regions necessary for particular aspects of processing. In more practical terms, we can use this information to monitor the disease progression, or to categorize a clinical entity into more homogeneous groups, which can be meaningful if such subgroups express differences in prognosis or response to various treatments.

## Discussion

We have described neuroinformatics technologies at 1 mm anatomical scale based on high-throughput 3D functional and structural imaging technologies of the human brain. The core is the conversion of functional and structural imagery into their high-dimensional neuroinformatic representations index containing O(1000–10,000) discriminating dimensions. The pipeline is based on advanced image analysis coupled to digital knowledge representations in the form of dense atlases of the human brain at gross anatomical scale. We demonstrate the integration of these high-dimensional representations with machine learning methods.

The neuroinformatics pipeline is used to examine cross-sectional and personalized analyses of neuropsychiatric illnesses in clinical applications as well as longitudinal studies. We have demonstrated the use of high-throughput machine learning methods for supporting (i) cross-sectional image analysis to evaluate the health status of individual subjects with respect to the population data, (ii) integration of image and non-image information for diagnosis and prognosis.

### Conflict of interest statement

Susumu Mori and Michael I. Miller own Anatomy Works, with Susumu Mori serving as its CEO. This arrangement is being managed by Johns Hopkins University in accordance with its conflict of interest policies. The other authors declare that the research was conducted in the absence of any commercial or financial relationships that could be construed as a potential conflict of interest.
